# Serum NY‐ESO‐1 and p53 antibodies as useful tumor markers in gastric cancer

**DOI:** 10.1002/ags3.12757

**Published:** 2023-11-20

**Authors:** Junji Kawada, Takuro Saito, Yukinori Kurokawa, Ryohei Kawabata, Atsushi Takeno, Tomohira Takeoka, Yohei Nose, Hisashi Wada, Hidetoshi Eguchi, Yuichiro Doki, Osaka University Clinical Research Group for Gastroenterological Study

**Affiliations:** ^1^ Department of Gastroenterological Surgery Osaka General Medical Center Osaka Japan; ^2^ Department of Surgery Kaizuka City Hospital Osaka Japan; ^3^ Department of Gastroenterological Surgery Osaka University Graduate School of Medicine Suita Japan; ^4^ Department of Surgery Osaka Rosai Hospital Sakai Japan; ^5^ Department of Surgery Kansai Rosai Hospital Amagasaki Japan; ^6^ Department of Surgery Hyogo Prefectural Nishinomiya Hospital Nishinomiya Japan; ^7^ Department of Clinical Research in Tumor Immunology Osaka University Graduate School of Medicine Osaka Japan

**Keywords:** antibody response, gastric cancer, NY‐ESO‐1, p53, tumor marker

## Abstract

**Purpose:**

The NY‐ESO‐1 antigen is highly immunogenic and often spontaneously induces an immune response in patients with cancer. We conducted a large‐scale multicenter cohort study to investigate the utility of serum NY‐ESO‐1 and p53 antibodies as predictive markers for the postoperative recurrence of gastric cancer. Here, we examined the usefulness of pre‐treatment NY‐ESO‐1 and p53 antibodies as tumor markers for the diagnosis of gastric cancer in combination with carcinoembryonic antigen (CEA) and carbohydrate antigen 19‐9 (CA19‐9).

**Methods:**

A total of 1031 patients with cT3‐4 gastric cancer were enrolled in the study. NY‐ESO‐1 and p53 antibodies were assessed prior to treatment. The positivity of NY‐ESO‐1 and p53 antibodies, CEA, and CA19‐9 was evaluated before treatment.

**Results:**

Serum NY‐ESO‐1 and p53 antibodies were positive in 12.6% and 18.1% of the patients, respectively. Positive NY‐ESO‐1 antibody response was correlated with male gender, higher cStage, and upper tumor location. However, a positive p53 antibody response was not associated with tumor factors. The combination of NY‐ESO‐1 or p53 antibody response with CEA and CA19‐9, or the 4‐factors, was positive in 45.1%, 49.6%, and 53.8% of patients, respectively. Moreover, the 4‐factor combination was able to detect >60% of cStage III‐IV diseases, which was 14% higher than that with the combination of CEA and CA19‐9.

**Conclusion:**

The combination of NY‐ESO‐1 and p53 antibody responses to CEA and CA19‐9 increases the diagnostic accuracy of gastric cancer. Serum NY‐ESO‐1 and p53 antibodies may be useful tumor markers for gastric cancer.

## Graphical abstract

Serum NY‐ESO‐1 and p53 antibodies were evaluated in 1,000 patients with gastric cancer before treatment. The combination of NY‐ESO‐1 and p53 antibody responses to CEA and CA19‐9 enhanced the diagnostic accuracy of gastric cancer. Serum NY‐ESO‐1 and p53 antibodies may be useful as tumor markers for gastric cancer.

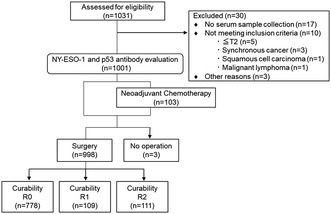

## 1 INTRODUCTION

Gastric cancer is a malignant tumor with a high mortality rate in Japan. The standard treatment for advanced gastric cancer is surgery, followed by adjuvant chemotherapy,^1^, ^2^ but patients with gastric cancer exhibit high relapse rates, even after curative surgery.^3^ Therefore, early detection of gastric cancer is important for effective treatment. Carcinoembryonic antigen (CEA) and carbohydrate antigen 19‐9 (CA19‐9) are general tumor markers for gastric cancer and are evaluated before treatment in Japan.^4^, ^5^ However, these serum markers were found to be positive in only 20%–60% of all cases, and 5% of CEA‐positive cases were false‐positive due to smoking, endometriosis, aging, or other factors.^6^ Thus, the development of a novel marker with higher diagnostic value for gastric cancer, or a combination of these markers, is urgently needed.

NY‐ESO‐1 antigen was originally identified in esophageal cancer by serological expression cloning (SEREX) using autologous patient serum.^7^, ^8^ The NY‐ESO‐1 antigen induces a strong immune response, and tumors expressing the NY‐ESO‐1 antigen often spontaneously exhibit humoral and cellular immunological responses.^9^, ^10^, ^11^ Therefore, we focused on the high prevalence of the NY‐ESO‐1 spontaneous antibody response in cancers and assumed that the antibody responses can be used as a tumor marker in gastric cancer.^12^ Several reports have shown the positivity of serum NY‐ESO‐1 antibody in gastric cancer, which was positive in 9.7% to 11.1% of patients,^13^ but these were retrospective studies with small sample sizes.

The serum p53 antibody is the only antibody‐based biomarker for clinical use in esophageal, colorectal, and breast cancers in Japan. The p53 protein is known as a driver mutation in several malignancies, and these mutant p53 proteins can induce p53 antibodies. In previous reports, p53 antibody was positive in 31%, 30%, and 16% of patients with esophageal cancer, colorectal cancer, and gastric cancer, respectively.^14^ High p53 antibody levels have been reported to be associated with poor prognosis in patients with esophageal cancer.^15^ However, the utility of p53 antibody as a tumor marker in gastric cancer has not been well‐investigated.

A large‐scale multicenter cohort study was conducted to investigate the utility of serum NY‐ESO‐1 and p53 antibodies as predictive markers for the postoperative recurrence of gastric cancer. The expression of NY‐ESO‐1 and p53 antibodies was evaluated in 1000 patients before treatment. In this study, we examined the utility of pre‐treatment with NY‐ESO‐1 and p53 antibodies as tumor markers for gastric cancer detection in combination with the common tumor markers, CEA and CA19‐9. Since NY‐ESO‐1 antibodies are produced in the immune response against malignant tumors expressing the NY‐ESO‐1 antigen, the NY‐ESO‐1 antibody may be a new tumor marker with a completely different mechanism from CEA and CA19‐9, which are tumor expression factors. Moreover, high therapeutic efficacy of immune checkpoint inhibitors (ICIs) had been reported in patients with positive NY‐ESO‐1 antibody responses in some malignancies.^16^, ^17^, ^18^, ^19^ Thus, the detection of NY‐ESO‐1 antibodies may be useful in identifying targets that are likely to respond favorably to ICIs. Although the positivity of NY‐ESO‐1 antibodies had been assessed in various types of cancers, most was reported with small cohorts. Therefore, this is the first report to evaluate the utility of NY‐ESO‐1 and p53 antibodies combined with CEA and CA19‐9 as tumor markers in more than 1000 patients with gastric cancer.

## 2 MATERIALS AND METHODS

### 2.1 Study design and participants

A multicenter, prospective cohort study was conducted in 21 Japanese institutions to assess the utility of postoperative serum NY‐ESO‐1 or p53 antibody positivity in predicting postoperative recurrence of gastric cancer. The study was organized by the Osaka University Clinical Research Group for Gastroenterological Study and was performed between January 1, 2012, and December 31, 2021. The inclusion criteria were as follows: newly diagnosed gastric cancer that can be curatively resected; histologically confirmed gastric adenocarcinoma; cT3 (SS)‐cT4b (SI); no clinical distant metastasis (H0, P0, M0); age 20–90 years; and Eastern Cooperative Oncology Group performance status 0–2. Patients with distant metastases were excluded; however, those with cStage IV disease due to resectable disease after chemotherapy were included. Clinical staging was evaluated according to the Japanese Classification of Gastric Cancer, 14th edition (JCGC), by endoscopy and CT imaging according to each institution's criteria. The exclusion criterion was active synchronous cancer, except carcinoma in situ. The study protocol was approved by the institutional review boards of all participating institutions belonging to the Clinical Study Group of Osaka University before the start of the study. All patients provided written informed consent prior to enrollment. This trial was registered with the UMIN‐CTR (number UMIN00007925).

### 2.2 Assessment of serum NY‐ESO‐1 and p53 antibodies

Serum samples were collected from patients and stored before surgery or chemotherapy. The NY‐ESO‐1 antibody was assessed using an enzyme‐linked immunosorbent assay (ELISA), as previously reported.^12^ Serum NY‐ESO‐1 antibody levels were assessed using optical density (O.D.) values. Examinations were performed to simultaneously evaluate as many samples as possible. The cut‐off value was defined as an O.D. value of 0.5 at a dilution of 1:100, which was the upper limit of the mean + 2 × standard deviation values from 50 healthy donors. The p53 antibody was assessed using ELISA (MESACUP anti‐p53 Test; Medical and Biological Laboratories Co. Ltd., Nagoya, Japan).^20^ The cut‐off value of the p53 antibody response was set at 1.3 U/mL.

### 2.3 Clinical information

Clinicopathological information, such as tumor stage, histological diagnosis, and blood test results, was collected from relevant patient databases. Serum CEA and CA19‐9 and other blood tests were evaluated using normal clinical laboratory tests. The cut‐off values were 5.0 ng/mL for CEA and 37 U/mL for CA19‐9.

### 2.4 Endpoints

The primary endpoint was the percentage of patients with postoperative recurrence based on the positivity of serum NY‐ESO‐1 or p53 antibody responses at 12 months after surgery in patients with positive preoperative NY‐ESO‐1 or p53 antibody responses. The secondary endpoints were the preoperative positive rates of serum NY‐ESO‐1 and p53 antibodies, correlation between preoperative NY‐ESO‐1 or p53 antibodies and gastric cancer prognosis, positive rates of tumor NY‐ESO1 and p53 antigen expression, and correlation between tumor NY‐ESO1 or p53 antigen expression and gastric cancer prognosis. Additionally, a sub‐analysis was conducted to investigate the combined positivity of tumor makers with NY‐ESO‐1 and p53 antibodies, CEA, and CA19‐9 before treatment.

### 2.5 Statistics

To determine the differences between the two groups, the chi‐square test was used for categorical variables and the Mann–Whitney U test was used for continuous variables. Statistical significance was set at *p* < 0.05. Statistical analyses were performed using the JMP Pro software version 10.0.0 (SAS Institute, Cary, NC, USA).

## 3 RESULTS

### 3.1 Study population

**FIGURE 1:**
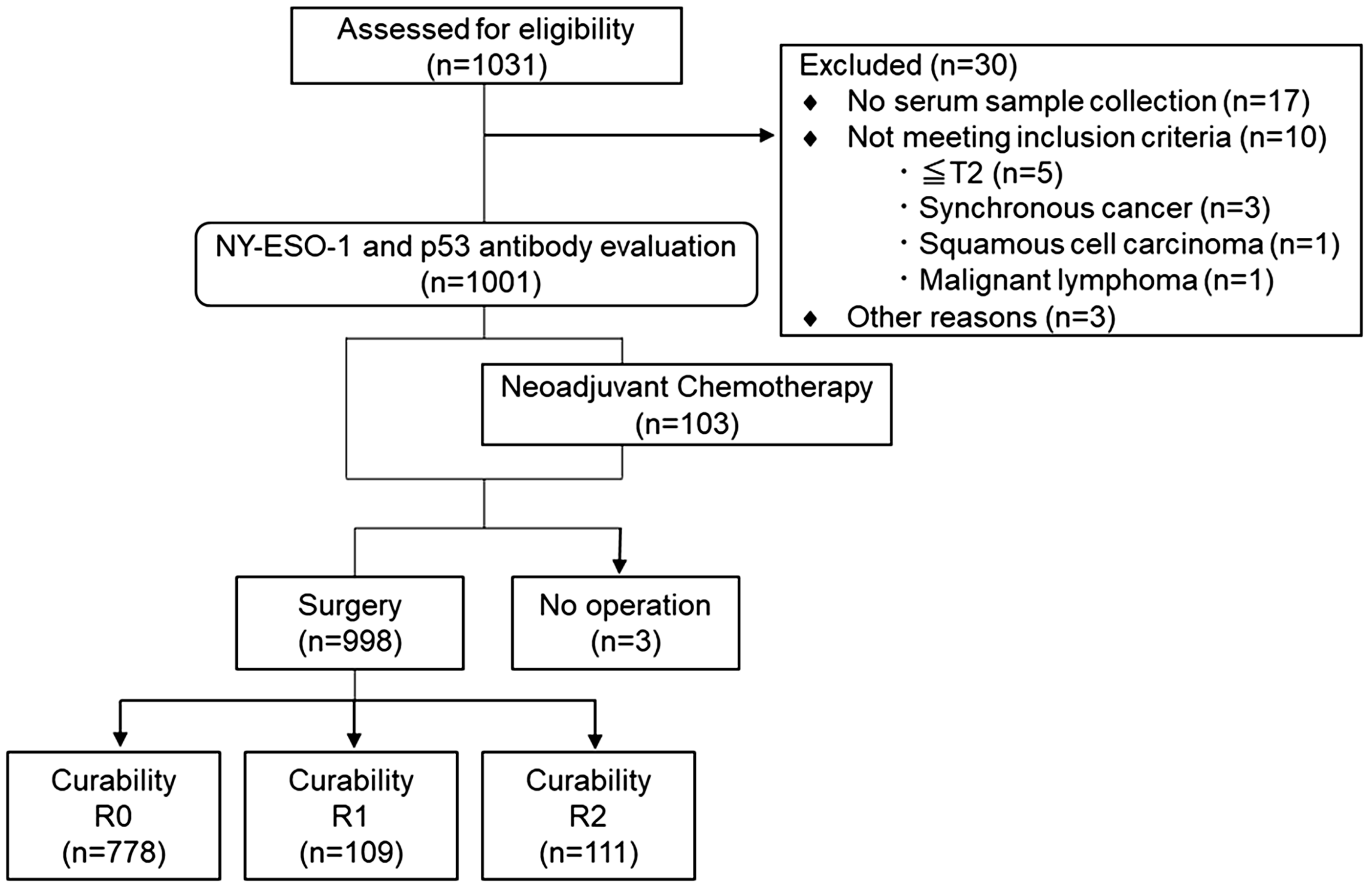
Schematic of the study design.

From January 2012 to March 2018, 1031 patients with cT3‐4 gastric cancer were evaluated for eligibility, of whom 30 did not meet the inclusion criteria (Figure 1). The remaining 1001 patients were evaluated for serum NY‐ESO‐1 and p53 antibodies. One hundred and three patients underwent neoadjuvant chemotherapy, 998 underwent surgery, and 778 underwent R0 resection.

### 3.2 The evaluation of NY‐ESO‐1 antibody and p53 antibody responses

Of the 1001 patients, serum NY‐ESO‐1 and p53 antibodies were positive in 126 (12.6%) and 181 (18.1%) patients, respectively (Table 1). Serum NY‐ESO‐1 antibody was positive in 47 (9.7%) of 486 patients with cStage II, 74 (15.4%) of 481 with cStage III, and five (14.7%) of 34 with cStage IV. Serum p53 antibody was positive in 85 (17.5%) of 486 patients with cStage II, 89 (18.5%) of 481 with cStage III, and seven (20.6%) of 34 with cStage IV. CEA and CA19‐9 levels were positive in 253 (25.3%) and 227 (22.7%) patients, respectively. The positivity for CEA and CA19‐9 increased as cStages increased.

**TABLE 1:** Positive frequencies of NY‐ESO‐1 antibody, p53 antibody, CEA, and CA19‐9 according to cStage in gastric cancer.

cStage	Total	NY‐ESO‐1 antibody	p53 antibody	CEA	CA19‐9
II	486	47 (9.7%)	85 (17.5%)	98 (20.3%)	75 (15.5%)
III	481	74 (15.4%)	89 (18.5%)	141 (29.3%)	136 (28.3%)
IV	34	5 (14.7%)	7 (20.6%)	14 (41.2%)	16 (47.1%)
II + III + IV	1001	126 (12.6%)	181 (18.1%)	253 (25.3%)	227 (22.7%)

*Note*: Data were lacking in two patients for CEA and three for CA19‐9.

### 3.3 The correlation of NY‐ESO‐1 antibody and p53 antibody response with clinicopathological factors

Positive NY‐ESO‐1 antibody response was correlated with older age (*p* = 0.012), male sex (*p* = 0.001), higher cN stage (*p* = 0.015), higher cStage (*p* = 0.007), tumor in upper location (*p* = 0.017), and differentiated histological type (*p* = 0.028) (Table 2). Positive p53 antibody response was not associated with any tumor factor. Moreover, positive NY‐ESO‐1 antibody response was associated with lower albumin levels (*p* = 0.024) and tended to be associated with higher white blood cell (WBC) counts (*p* = 0.059) and lower lymphocyte counts (*p* = 0.087) (Table 3). Both NY‐ESO‐1 and p53 antibody responses were correlated with CEA positivity (*p* < 0.0001 and *p* = 0.022, respectively) and tended to correlate with each other (*p* = 0.074).

**TABLE 2:** Clinicopathological factors based on NY‐ESO‐1 and p53 antibody status.

	NY‐ESO‐1 antibody (−)	NY‐ESO‐1 antibody (+)	*p* value	p53 antibody (−)	p53 antibody (+)	*p* value
	(*n* = 875)	(*n* = 126)		(*n* = 820)	(*n* = 181)	
Age, years	72 (34–90)	74 (41–90)	0.012	72 (34–90)	73 (40–90)	0.59
Gender						
Male	599 (68.5%)	105 (83.3%)	0.001	564 (68.8%)	140 (73.3%)	0.022
Female	276 (31.5%)	21 (16.7%)		256 (31.2%)	41 (22.7%)	
BMI (g/m^2^)	22.1 (12.9–36.6)	21.9 (13.8–30.6)	0.60	22.1 (12.9–36.6)	22.3 (13.8–34.8)	0.41
Performance status						
0–1	803 (91.8%)	117 (92.9%)	0.68	755 (92.1%)	165 (91.2%)	0.68
2–4	72 (8.2%)	9 (7.1%)		65 (7.9%)	16 (8.8%)	
cT						
3	430 (49.1%)	60 (47.6%)	0.75	406 (49.5%)	84 (46.4%)	0.45
4	445 (50.9%)	66 (52.4%)		414 (50.5%)	97 (53.6%)	
cN						
0–1	589 (67.3%)	71 (56.3%)	0.015	452 (66.1%)	118 (65.2%)	0.82
2–3	286 (32.7%)	55 (43.7%)		278 (33.9%)	63 (34.8%)	
cM						
0	846 (96.7%)	121 (96.0%)	0.71	793 (96.7%)	174 (96.1%)	0.70
1	29 (3.3%)	5 (4.0%)		27 (3.3%)	7 (3.9%)	
cStage						
2	439 (50.2%)	47 (37.3%)	0.007	401 (48.9%)	85 (47.0%)	0.64
3–4	436 (49.8%)	79 (62.7%)		419 (51.1%)	96 (53.0%)	
Tumor location						
Lower–middle	663 (75.8%)	83 (65.9%)	0.017	614 (74.9%)	132 (72.9%)	0.59
Upper	212 (24.2%)	43 (30.2%)		206 (25.1%)	49(27.1%)	
Histological type^a^						
Differentiated	396 (45.3%)	73 (57.9%)	0.028	378 (46.1%)	91 (50.3%)	0.41
Undifferentiated, Others	428 (48.9%)	47 (37.3%)		397 (48.4%)	78 (43.1%)	
No dissection	51 (5.8%)	6 (4.8%)		45 (5.5%)	12 (6.6%)	
Neoadjuvant chemotherapy						
No	783 (89.5%)	115 (91.3%)	0.54	738 (90.0%)	160 (88.4%)	0.52
Yes	92 (10.5%)	11 (8.7%)		82 (10.0%)	21 (11.6%)	
Surgical curability						
R0	680 (77.7%)	98 (77.8%)	0.55	647 (78.9%)	131 (72.4%)	0.14
R1–2	193 (22.1%)	27 (21.4%)		171 (20.9%)	49 (27.1%)	
No operation	2 (0.2%)	1 (0.8%)		2 (0.2%)	1 (0.6%)	

*Note*: Data are shown as number of patients (%) or median (min‐max). TNM stage was classified according to the 14th edition of the Japanese Classification of Gastric Carcinoma.

^a^ Differentiated: pap/tub1/tub2, undifferentiated: por/sig, others: muc/NEC/carcinoma with lymphoid stroma/hepatoid adenocarcinoma/undifferentiated carcinoma.

**TABLE 3:** Correlation between blood markers and NY‐ESO‐1 and p53 antibody status.

	NY‐ESO‐1 antibody (−)	NY‐ESO‐1 antibody (+)	*p* value	p53 antibody (−)	p53 antibody (+)	*p* value
WBC (g/dL)						
<6000	383 (43.8%)	48 (34.9%)	0.059	354 (43.2%)	73 (40.3%)	0.48
≧6000	491 (56.2%)	82 (65.1%)		465 (56.8%)	108 (59.7%)	
Lymphocytes (g/dL)						
<1500	416 (47.8%)	70 (56.0%)	0.087	391 (48.0%)	95 (52.8%)	0.24
≧1500	454 (52.2%)	55 (44.0%)		424 (52.0%)	85 (47.2%)	
Hb (g/dL)						
<12.0	404 (46.3%)	55 (52.8%)	0.17	382 (46.8%)	88 (48.6%)	0.65
≧12.0	469 (53.7%)	59 (47.2%)		435 (53.2%)	93 (51.4%)	
Alb (g/dL)						
<3.8	383 (44.0%)	69 (54.8%)	0.024	372 (45.6%)	80 (44.2%)	0.72
≧3.8	487 (56.0%)	57 (45.2%)		443 (54.4%)	101 (55.8%)	
CEA (dL/mL)						
−	669 (76.7%)	76 (60.3%)	<0.0001	622 (76.1%)	123 (68.0%)	0.022
+	203 (23.3%)	50 (39.7%)		195 (23.9%)	58 (32.0%)	
CA19‐9 (U/mL)						
−	680 (78.0%)	91 (72.2%)	0.15	631 (77.2%)	140 (77.3%)	0.97
+	192 (22.0%)	35 (27.8%)		186 (22.8%)	41 (22.7%)	
p53 antibody (IU/mL)						
−	724 (82.7%)	96 (76.2%)	0.074			
+	151 (17.3%)	30 (23.8%)				

*Note*: Data were lacking for one patient for WBC, two for CEA, and three for CA19‐9.

Abbreviations: Hb, hemoglobin; WBC, white blood cell.

### 3.4 The diagnostic accuracy with the combination of NY‐ESO‐1 and p53 antibody response, CEA and CA19‐9

Next, we examined whether the combination of NY‐ESO‐1 and p53 antibody responses to CEA and CA19‐9 would increase the accuracy of gastric cancer diagnosis (Table 4). The combination of CEA and CA19‐9 was positive in 392 (39.2%) patients. In contrast, the combination of NY‐ESO‐1 or p53 antibody response with CEA and CA19‐9 was positive in 451 (45.1%) and 495 (49.6%) patients, respectively, and the 4‐factor combination was positive in 537 patients (53.8%). In particular, the 4‐factor combination detected 61.2% of cStage III–IV diseases.

**TABLE 4:** Positive frequencies with a combination of NY‐ESO‐1 antibody, p53 antibody, CEA, and CA19‐9 according to cStage.

cStage	Total	CEA	NY‐ESO‐1 antibody	p53 antibody	NY‐ESO‐1‐antibody
					p53 antibody
			CEA	CEA	CEA
		CA19‐9	CA19‐9	CA19‐9	CA19‐9
II	486	149 (30.7%)	173 (35.6%)	204 (42.2%)	222 (46.0%)
III	481	222 (46.2%)	255 (53.0%)	268 (55.7%)	290 (60.3%)
IV	34	21 (61.8%)	23 (67.6%)	23 (67.6%)	25 (73.5%)
III + IV	515	243 (47.2%)	278 (54.0%)	291(56.5%)	315 (61.2%)
II + III + IV	1001	392 (39.2%)	451 (45.1%)	495 (49.6%)	537 (53.8%)

*Note*: Data were lacking in two patients for CEA and three for CA19‐9.

## 4 DISCUSSION

Our study revealed that serum NY‐ESO‐1 and p53 antibodies were positive in 12.6% and 18.1% of patients with gastric cancer, respectively, and the combination of NY‐ESO‐1 and p53 antibody responses to general tumor markers CEA and CA19‐9 increased the diagnostic accuracy of gastric cancer. The positivity rates were 45.1%, 49.6%, and 53.8% in the combination of NY‐ESO‐1 or p53 antibody response with CEA and CA19‐9, and the 4‐factor combination, respectively. In particular, the 4‐factor combination was able to detect more than 60% of cStage III‐IV diseases, which was 14% higher than the detection rate of the combination of CEA and CA19‐9.

The NY‐ESO‐1 gene is located in the Xq28 region of the X chromosome, and the NY‐ESO‐1 antigen is expressed in spermatogonial cells, primary spermatocytes, oocytes, the placenta, and various cancers.^21^ Specific demethylation of cancer testis gene promotor regions was shown to be responsible for induction of CT antigen expression in cancer.^22^, ^23^ Although the function of NY‐ESO‐1 is still unknown, the NY‐ESO‐1 antigen has strong immunogenicity and spontaneously elicits both humoral and cellular immunity.^24^ Serum NY‐ESO‐1 antibody has been detected in several types of cancers, including in 3.9%, 1.6%, 12.6%, 26.3%, and 9.3% of patients with esophageal,^11^ breast,^10^ bile duct,^25^ small cell lung,^26^ and non‐small cell lung cancers,^18^ respectively. However, these antibodies were not detected in healthy donors. Thus, the positivity of NY‐ESO‐1 antibodies indicates the existence of malignant tumors expressing the NY‐ESO‐1 antigen, though it's not limited to gastric cancer. In this study, NY‐ESO‐1 antibody was positive in 12.6% of patients with gastric cancer, which was consistent with a previous report.^12^, ^13^, ^27^ Furthermore, positivity was significantly higher in patients who were older, male, had higher cN stage, higher cStage, tumor in upper location, differentiated histological type, and lower albumin levels. The multivariate analysis revealed that male gender, cStage 3–4, and upper tumor location were significantly correlated with NY‐ESO‐1 positivity (Table S1). As for age and sex, a correlation between NY‐ESO‐1 antibody response and older age or male sex has been reported in patients with esophageal and gastric cancer.^13^ It has also been reported that the expression of other cancer testis antigens, MAGE‐1 and BAGE, was higher at metastatic sites than at primary sites in patients with melanoma,^28^, ^29^ suggesting that the NY‐ESO‐1 antigen may be highly expressed at metastatic sites, such as metastatic lymph nodes, which may be the reason for the presence of serum NY‐ESO‐1 antibody in patients with more advanced gastric cancer. Lower albumin levels were associated with NY‐ESO‐1 positivity, possibly because nutritional status deteriorates as gastric cancer progresses. Furthermore, positive NY‐ESO‐1 antibody responses tended to be associated with higher WBC and lymphocyte counts. Since it is expected that antibody responses are more likely to occur in patients with better immune conditions, this result may reflect the fact that immune responses are more likely to occur in patients with higher WBC and lymphocyte counts. While a large cohort of healthy donors is lacking to directly examine the substantial correlation between NY‐ESO‐1 and the detection of gastric cancer regardless of these clinicopathological factors, the assessment of NY‐ESO‐1 antibodies could be meaningful for the identification of malignant tumors expressing the NY‐ESO‐1 antigen.

p53 is an important tumor suppressor gene, and missense mutations usually inactivate its tumor‐suppressing activity, simultaneously generating oncogenic mutant p53 proteins.^30^ The p53 antigen has strong immunogenicity and spontaneously elicits immune responses, and the serum p53 antibody was reported to be positive in 32.9%, 15%, and 4.5% of patients with esophageal cancer, gastric cancer, and hepatocellular carcinoma, respectively.^13^ In this study, the positivity of the p53 antibody was 18.1% and was significantly higher in male patients, which is consistent with the results of a previous report.^13^ As for cellular immunity, only p53‐specific CD4^+^ T cell responses were induced, whereas both NY‐ESO‐1‐specific CD8^+^ and CD4^+^ T cell responses were induced in patients with cancer.^24^ Since normal cells have normal p53 protein levels, T cells against p53 antigen may be regulated in the thymus as a central tolerance. In other words, p53 antibody responses can be induced against both normal and mutant proteins, which may explain why the expression rates of p53 antibodies were the same even at earlier stages.

CEA and CA19‐9 are generally used as tumor markers for various types of cancers. CEA was positive in 23.0%, 25.6%, and 39.5% of patients, while CA19‐9 was positive in 19.9%, 32.2%, and 44.7% of patients with Stage II, III, and IV gastric cancers, respectively.^31^ Other tumor markers reported in gastric cancer are CA72‐4, alpha‐fetoprotein, (AFP), CA125, and sialyl Tn antigen (STN). CA72‐4 positivity was the highest among these markers, and the positivity rates were 15.6%, 36.7%, and 49.6% in Stage II, III, and IV gastric cancers, respectively.^31^ The combination of CA72‐4 with CEA and CA19‐9 was 14% higher than that of CEA and CA19‐9.^32^ Regarding the combination of markers other than CEA and CA19‐9, the combination of CA72‐4 and CA125 was positive in 68.0% of patients with gastric cancer with peritoneal metastasis.^33^ In this study, the combination of NY‐ESO‐1 and p53 antibodies with CEA and CA19‐9 was positive in 61.2% of cStage III‐IV diseases, which was 14% higher than that with the combination of CEA and CA19‐9. Therefore, NY‐ESO‐1 and p53 antibodies may be promising candidate tumor markers for the diagnosis of gastric cancer in combination with CEA and CA19‐9.

Recently, ICIs have been used to treat various cancer types. The presence of anti‐tumor antigen‐specific T cells is recognized as crucial for the therapeutic efficacy of ICIs.^16^ In the context of cancer‐testis antigens, which are a subset of cancer antigens, it is possible that T cells specific to cancer‐testis antigens target tumors that express these antigens during ICI treatment. Indeed, there have been reports indicating that melanoma patients exhibiting a positive immune response to NY‐ESO‐1 showed favorable responses to anti‐CTLA‐4 therapy.^17^ Other studies have demonstrated that lung adenocarcinoma patients with serum NY‐ESO‐1 antibody responses exhibited improved clinical outcomes with anti‐PD‐1 therapy, compared to those without antibody responses.^18^, ^19^ Hence, gastric cancer patients with NY‐ESO‐1 antibody responses may respond to ICIs, suggesting the significance of evaluating NY‐ESO‐1 antibody responses to identify promising candidates for ICI treatment.

This study had several limitations. First, as this study enrolled patients with cT3 or higher gastric cancer, there was no information on early gastric cancer. However, recurrence is highly prevalent in advanced diseases, and information on advanced gastric cancers could be more meaningful. Second, the present results were confirmed with the discovery cohort only, and the utility of serum NY‐ESO‐1 and p53 antibodies as tumor markers in gastric cancer needs to be examined in other cohorts.

## 5 CONCLUSION

In conclusion, the combination of NY‐ESO‐1 and p53 antibody responses to CEA and CA19‐9 increased the diagnostic accuracy for gastric cancer; thus, NY‐ESO‐1 and p53 antibodies may be useful tumor markers for gastric cancer. Additionally, these patients with positive NY‐ESO‐1 antibody responses may represent promising candidates for ICI treatment. Since this study only included a discovery cohort, future prospective studies are needed to confirm the usefulness of serum NY‐ESO‐1 and p53 antibodies as tumor markers in gastric cancer.

## AUTHOR CONTRIBUTIONS

Takuro Saito designed the protocol. Junji Kawada and Takuro Saito collected and analyzed the data. Yuichiro Doki was the main investigator. Junji Kawada drafted the article. Takuro Saito, Yukinori Kurokawa, Hisashi Wada, and Yuichiro Doki helped finalize the article. All other authors participated in patient recruitment. All authors have read and approved the final article.

## FUNDING INFORMATION

The Supporting Center for Clinical Research and Education (SCCRE) partly supported this study and served as data manager.

## CONFLICT OF INTEREST STATEMENT

This study was partly funded by the Supporting Center for Clinical Research and Education (SCCRE). Yukinori Kurokawa and Yuichiro Doki are the editorial members of *Annals of Gastroenterological Surgery*.

## ETHICAL APPROVAL

All procedures performed in studies involving human participants were in accordance with the ethical standards of the institutional research committee and with the 1964 Helsinki Declaration and its later amendments. Informed consent was obtained from all patients prior to their inclusion in the study.

## Supporting information


Table S1.
