# Supplementary material for: Serum NY‐ESO‐1 and p53 antibodies as useful tumor markers in gastric cancer
Source: Ann Gastroenterol Surg. 2023 Nov 20;8(2):243–50. doi: 10.1002/ags3.12757 (PMC10914697; doi:10.1002/ags3.12757)
Supplement: Supplementary file 1 — Table S1. [file AGS3-8-243-s001.docx]

**Supplementary Table 1.** Univariate and multivariate analyses for positive NY-ESO-1 antibody responses

|  | Univariate analysis HR (95% CI) | *P* value | Multivariate analysis HR (95% CI) | *P* value |
| --- | --- | --- | --- | --- |
| Age (Years) (≥70 vs. <70) | 1.49 (1.01-2.21) | 0.046 | 1.47 (0.96-2.24) | 0.076 |
| Gender (Male vs. Female) | 2.30 (1.41-3.76) | 0.001 | 2.23 (1.33-3.73) | 0.002 |
| cStage (3, 4 vs. 2) | 1.69 (1.15-2.49) | 0.007 | 1.66 (1.10-2.51) | 0.016 |
| Tumor location  (Upper vs. Lower, Middle) | 1.05 (1.01-1.10) | 0.018 | 1.06 (1.01-1.11) | 0.016 |
| Histological type  (Differentiated vs. Others) | 1.68 (1.14-2.48) | 0.009 | 1.49 (0.99-2.22) | 0.052 |
| Alb (g/dl)  (＜3.8 vs. ≧3.8) | 1.54 (1.06-2.24) | 0.018 | 1.37 (0.91-2.07) | 0.135 |

HR, hazard ratio; CI, confidence interval
